# Changes over time in inflammatory and structural lesions at the sacroiliac joint in children with spondyloarthritis exposed and unexposed to tumor necrosis factor inhibitor

**DOI:** 10.1186/s12969-021-00647-6

**Published:** 2021-12-02

**Authors:** Timothy G. Brandon, Rui Xiao, Rosemary G. Peterson, Nancy A. Chauvin, Michael L. Francavilla, David M. Biko, Dax G. Rumsey, Matthew L. Stoll, Pamela F. Weiss

**Affiliations:** 1 Division of Rheumatology and Center for Pediatric Clinical Effectiveness at the Children’s Hospital of Philadelphia, Department of Pediatrics, Philadelphia, USA; 2 Department of Biostatistics, Epidemiology and Informatics, Perelman School of Medicine, University of Pennsylvania, Philadelphia, USA; 3 Division of Rheumatology at the Children’s Hospital of Philadelphia, Department of Pediatrics, Philadelphia, USA; 4 Department of Radiology at Penn State Health Milton S. Hershey Children’s Hospital, Hershey, PA USA; 5 Department of Radiology at the Children’s Hospital of Philadelphia and Department of Radiology, Perelman School of Medicine at the University of Pennsylvania, Philadelphia, USA; 6 Division of Pediatric Rheumatology, Department of Pediatrics, University of Alberta, Edmonton, Alberta Canada; 7 Division of Pediatric Rheumatology, Department of Pediatrics, University of Alabama at Birmingham, Birmingham, AL USA; 8 Department of Pediatrics, Division of Rheumatology and Center for Pediatric Clinical Effectiveness at the Children’s Hospital of Philadelphia and Center for Clinical Epidemiology and Biostatistics, Perelman School of Medicine at the University of Pennsylvania, 2716 South Street, Room 11121, Philadelphia, PA 19104 USA

**Keywords:** SACROILIAC JOINT, MAGNETIC RESONANCE IMAGING, PEDIATRICS, SPONDYOLARTHRITIS, DISEASE PROGRESSION, TUMOR NECROSIS FACTOR INHIBITORS

## Abstract

**Background:**

The objective of this work was to describe magnetic resonance imaging (MRI) changes over time in inflammatory and structural lesions at the sacroiliac joint (SIJ) in children with spondyloarthritis (SpA) exposed and unexposed to tumor necrosis factor inhibitor (TNFi).

**Methods:**

This was a retrospective, multicenter study of SpA patients with suspected or confirmed sacroiliitis who underwent at ≥2 pelvic MRI scans. Images were reviewed independently by 3 radiologists and scored for inflammatory and structural changes using the Spondyloarthritis Research Consortium of Canada (SPARCC) SIJ inflammation score (SIS) and structural score (SSS). Longitudinal, quantitative changes in patient MRI scans were measured using descriptive statistics and stratified by TNFi exposure. We used an average treatment effects (ATE) regression model to explore the average effect of TNFi exposure over time on inflammatory and structural lesions, adjusting for baseline lesion scores.

**Results:**

Forty-six subjects were evaluated using the SIS (*n* = 45) and SSS (*n* = 18). Median age at baseline imaging was 13.6 years, 63% were male and 71% were white. Twenty-three subjects (50%) were TNFi exposed between MRI studies. The median change in SIS in TNFi exposed and unexposed subjects with a baseline SIS ≥0 was − 20.7 and − 14.3, respectively (*p* = 0.09). Eleven (85%) TNFi exposed and 8 (89%) unexposed subjects with a baseline SIS ≥0 met the SIS minimal clinically important difference (MCID; ≥2.5). Using the ATE model adjusted for baseline SIS, the average effect of TNFi on SIS in patients with a baseline SIS ≥2 was − 14.5 (*p* < 0.01). Unadjusted erosion change score was significantly worse in TNFi unexposed versus exposed subjects (*p* = 0.03) but in the ATE model the effect of TNFi was not significant.

**Conclusion:**

This study quantitatively describes how lesions in the SIJs on MRI change over time in patients exposed to TNFi versus unexposed. Follow-up imaging in TNFi exposed patients showed greater improvement than the unexposed group by most metrics, some of which reached statistical significance. Surprisingly, a majority of TNFi unexposed children with a baseline SIS≥2 met the SIS MCID. Additional studies assessing the short and long-term effects of TNFi on inflammatory and structural changes in juvenile SpA are needed.

## Background

Juvenile spondyloarthritis (SpA) encompasses a group of heterogeneous conditions characterized by chronic inflammatory arthritis, enthesitis, inflammatory bowel disease, acute anterior uveitis, psoriasis, and HLA-B27 positivity. Up to 30 % of children with SpA develop sacroiliitis within several years of diagnosis [1]. If untreated, this can progress to ankylosing spondylitis (AS), an inflammatory disease that causes joint fusion and leads to permanent functional impairment [1–4] . Compared to adults with AS, children with SpA have worse functional outcomes, including sacroiliac radiographic scores and spinal deformity [5, 6]. While similarities exist between juvenile and adult SpA, there are distinct phenotypic differences that warrant specific focus on juvenile disease [7–11]. In comparison to adult-onset disease, juvenile-onset disease is associated with a higher prevalence of affected females [9], more peripheral disease [8, 11], less back pain [10], and less involvement of the spine [9, 11]. Additionally, several studies demonstrate that adult patients with juvenile-onset disease have more radiographic changes of the hips and significantly higher risk of hip replacement [7–9], making early recognition critical to preserve long-term functional outcomes.

In adults, TNF-inhibitor (TNFi) medications have been shown to help symptomatically and appear to lead to faster resolution of the inflammatory phase and start of tissue repair at sites of erosion [12–14]. It is hypothesized that TNFi medications may slow the progression toward new bone formation and ankylosis, although this has not yet been definitively proven. In children, the effectiveness of TNFi medications for axial disease and magnitude of response on MR imaging of the sacroiliac joints (SIJs) is not known. In a study by Bray et al of adolescents and young adults with sacroiliitis, SPARCC inflammation scores improved after TNFi exposure while structural scores did not; however, there was not a TNFi naïve comparison group [15]. Further characterization of the changes over time in in inflammatory and structural lesions at the SIJ in children with SpA exposed and unexposed to TNFi is needed.

MRI has become increasingly preferred as the primary imaging modality for evaluating disease in the sacroiliac joint due to its ability to detect inflammation prior to development of bony damage detectable by radiographs. Additionally, in children, radiographs have been shown to have high rates of misclassification of sacroiliitis using MRI as the reference standard with false positive radiographs occurring more frequently than false negative radiographs [16]. The Spondyloarthritis Research Consortium of Canada (SPARCC) sacroiliac inflammation (SIS) and structural scores (SSS) are validated measures to assess the progression of sacroiliitis on MRI and have been found to be both reliable and responsive to therapeutic intervention in adults [17–19]. Both have also shown to be reliable and feasible measures in children with suspected or confirmed juvenile SpA [20].

Characterization of the effect of biologic agents on the progression of sacroiliitis in children with SpA is critical to understanding the underlying disease pathophysiology and choosing the most effective treatments. The rarity of juvenile SpA limits the feasibility of randomized clinical trials and necessitates performing innovative observational studies. We aimed to evaluate the change in the SPARCC SIS and SSS lesions over time in TNFi-exposed versus TNFi-unexposed patients.

## Methods

This study’s protocol was reviewed and approved by the University of Alabama at Birmingham’s (IRB-160701011) and Children’s Hospital of Philadelphia’s (IRB 16–013015) Committees for the Protection of Human Subjects and the ethics committee at the University of Alberta (REB Pro00081651). Waivers of consent and HIPAA authorization were granted as procedures represented minimal risk to the subjects and did not adversely affect the rights and welfare of the subjects.

### Study population

This was a retrospective cohort study of children with SpA and suspected sacroiliitis who underwent at least two pelvic MRI scans spaced at least 12 weeks or 2 years apart for assessment of the SIS and SSS change scores, respectively, between January 2005 and July 2020. Studies were obtained at the treating physician’s discretion. Eligible cases were aged 0–19 years old at time of baseline imaging. Patients with ≥4 weeks of TNFi exposure prior to the first MRI were excluded. Pelvic MRI studies were performed at the Children’s Hospital of Philadelphia (Philadelphia, Pennsylvania, USA), Children’s of Alabama (Birmingham, Alabama, USA), Stollery Children’s Hospital (Edmonton, Alberta, Canada), and the Grey Nuns’ Hospital (Edmonton, Alberta, Canada). Since this was not a prospective protocolized study, imaging sequences were slightly different at the three participating institutions, but all studies were performed on high field-strength magnets (1.5 T or 3.0 T). As long as there was a semicoronal T1-weighted (T1W) and short tau inversion recovery (STIR) or other equivalent fluid-sensitive sequence of the sacroiliac joints, subtle differences in MRI protocols did not preclude SPARCC SIS or SSS scoring. Imaging studies that did not include either the fluid-sensitive or T1W coronal oblique sequence were excluded for evaluation of the SIS and SSS, respectively. Radiology archiving systems and clinic records were queried to identify potentially eligible subjects and abstract physical examination reports, disease activity measures, patient-reported pain and global assessment scales, and medication use in the period leading up to and between the MRIs.

### MRI evaluation

All cases were centrally collected, anonymized, and scored independently in pairs blinded to time point. Three radiologists (NC, MF, DB) with extensive experience in sacroiliac joint imaging used the online viewing and scoring system at www.CaREArthritis.com to score the Digital Imaging and Communication in Medicine (DICOM)-based cases. The radiologists scored the SPARCC SIS, SSS, or both depending on what MRI sequences were available. For patients with more than two studies, the first and second study were included in the analysis for SIS to provide the best estimate of rate of change in acute inflammation, while the first and last qualifying studies were included in the analysis for SSS to provide a sufficient time window for structural changes to accrue. Patients with an SIS of zero on both MRI scans were included because, clinically, axial arthritis was suspected and excluding those patients could disproportionately bias the results for the TNFi unexposed group away from a null finding.

The SIS divides each sacroiliac joint into quadrants and scores presence, depth, and intensity of bone marrow edema (BME) on STIR MRI sequences and has been demonstrated to be reliable and valid in the pediatric population for both status and change scores [20–22]. Six consecutive semicoronal slices through the cartilaginous portion of the joint are scored for BME (total score 0–72). The accepted minimal clinically important difference (MCID) in the SPARCC SIJ inflammation score is 2.5, established during a randomized, placebo-controlled clinical trial of adalimumab where participants reported their global evaluation of change on a scale of “much worse” to “much better” at each MRI visit [17]. A SPARCC SIS score of ≥2 was used as a surrogate for meeting the Assessment of Spondyloarthritis International Society (ASAS) definition of a positive MRI [23].

The SSS assesses a spectrum of structural lesions of the SIJ on MRI including erosion, backfill, fat metaplasia, and ankylosis on 5 consecutive slices through the cartilaginous part of the joint and has been shown to be reliable for both status and change scores [20, 22]. These components are scored 0–20 (backfill and ankylosis) or 0–40 (erosion and fat metaplasia). For the evaluation of pediatric cases, sclerosis is also included and scored 0–40 [20]. Standards of interpretation and terminology were established by having the radiologists view training modules and conduct calibration exercises using 30 reference cases as publicly available on www.CaREarthritis.org. All raters previously completed calibration exercises for both the SPARCC SIS and SSS, achieving acceptable reliability. For cases on which the 2 raters disagreed about the presence/absence of SIS or SSS components, the case was scored by a 3rd rater. Scores for the SIS and all SSS components were averaged across the two raters who agreed about the presence or absence of a lesion to provide a final score for each case.

### Analysis

Study cohort demographics, clinical features, and component scores assigned by radiologists were summarized using standard descriptive statistics. Intraclass correlation coefficients (ICCs) were calculated to assess interrater reliability using two-way mixed-effects models measuring absolute agreement in scoring by radiologists. Interpretation of ICCs were as follows: ICC < 0.40 was poor, 0.40 ≤ ICC < 0.75 was fair to good, and ICC ≥ 0.75 was excellent [24]. These interpretation thresholds were used in the evaluation of the SPARCC SIS and SSS status and change scores in pediatrics [20–22]. Spearman’s correlation was used to assess the relationship of change in pain score with change in SIS.

We used regression adjustment in an average treatment effects (ATE) model, allowing for a comparison of the sample mean difference between those treated and those untreated, to explore the average effect of TNFi exposure between scans on inflammatory and structural lesions, adjusting for baseline lesion scores. Subjects were considered TNFi exposed if they were treated with a TNFi for 90 days or more between the two MRI studies. To test for robustness of our choice of exposure window, sensitivity analyses were performed with different windows of TNFi treatment prior to the second MRI (60, 90, 120, and 180 days). Statistical analyses were conducted using Stata 14.2 (StataCorp. 2015, *Stata Statistical Software: Release 14.* College Station, TX: StataCorp LP).

## Results

### Subjects

A total of 57 unique patients from three tertiary care centers were available for evaluation. After exclusion of 11 patients due to TNFi exposure ≥4 weeks prior to baseline imaging, 46 eligible patients were included in the analysis. Demographic and clinical characteristics of the patients at the time of baseline imaging are shown in Table 1. The majority of patients in this cohort were diagnosed with enthesitis-related arthritis (89%). The remaining patients were diagnosed with either psoriatic arthritis, undifferentiated arthritis, or inflammatory bowel disease-associated arthritis. SIS was assessed in 45 cases and SSS was assessed in 18 cases. The median age at the time of baseline imaging was 13.6 years (IQR: 11.4–15.4), 63% were male and 71% were white. The median duration of disease at the time of baseline imaging was 11.3 months (IQR: 3.7–23.7) and time between imaging studies included for assessment of change in the SIS and SSS were 14.5 (IQR: 6.0–25.3) and 46.1 (IQR: 27.4–52.7) months, respectively. Twenty-three subjects (50%) were TNFi-exposed between the two MRI scans. Four (9%), 14 (30%) and 14 (30%) received infliximab, etanercept or adalimumab respectively; six subjects received more than one TNFi sequentially between SIS-eligible MRI studies and six subjects received more than one TNFi between SSS-eligible MRI studies. One subject was considered TNFi unexposed by study exposure definition but received 67 days of TNFi between MRI studies. There were four patients exposed to a non-TNFi biologic disease-modifying antirheumatic drug (bDMARD); all were also TNFi exposed. Three of four started the TNFi first and, of those, two did not start the non-TNFi until after the change in inflammation follow-up assessment period (post-MRI 2). One of the four patients only had sequences available to evaluate the SIS.

**Table 1:** Subject characteristics

	All subjects	TNFi exposed	TNFi unexposed
	(***N*** = 46)	(***N*** = 23)	(***N*** = 23)
Age, years (Median, IQR)	13.6 (11.4, 15.4)	13.1 (11.4, 15.3)	13.9 (11.4, 15.4)
Sex, male	29 (63.04%)	13 (56.52%)	16 (69.57%)
Race, white	30 (71.43%) [*N* = 42]	16 (72.73%) [*N* = 22]	14 (70.00%) [*N* = 20]
HLA-B27 positive	23 (50.00%)	14 (60.87%)	9 (39.13%)
Hip arthritis	12 (26.09%)	8 (34.78%)	4 (17.39%)
Lower back pain	27 (58.70%)	12 (52.17%)	15 (65.22%)
Morning stiffness (> 15 min)	20 (45.45%) [*N* = 44]	10 (45.45%) [*N* = 22]	10 (45.45%) [*N* = 22]
Disease duration, months (Median, IQR)	11.3 (3.7, 23.7)	9.5 (2.9, 20.3)	12.0 (3.9, 36.2)
Active peripheral joint count (Median, IQR)	0.0 (0.0, 2.0)	0.0 (0.0, 2.0)	0.0 (0.0, 2.0)
Tender entheses (Median, IQR)	1.5 (0.0, 4.0)	0.0 (0.0, 5.0)	2.0 (0.0, 4.0)
Physician global (0–10; Median, IQR)	2.0 (2.0, 4.0) [*N* = 34]	2.0 (2.0, 4.0) [*N* = 21]	2.0 (1.0, 3.0) [*N* = 13]
Patient global (0–10; Median, IQR)	4.3 (2.0, 6.0) [*N* = 30]	5.0 (3.0, 6.0) [*N* = 19]	2.0 (1.0, 7.0) [*N* = 11]
Patient pain (0–10; Median, IQR)	5.0 (1.6, 7.0) [*N* = 30]	5.6 (2.0, 7.0) [*N* = 19]	3.0 (1.0, 7.0) [*N* = 11]
csDMARD use during follow-up	17 (36.96%)	8 (34.78%)	9 (39.13%)
Non-TNFi bDMARD use during follow-up*	4 (8.70%)	4 (17.39%)	0 (0.00%)
NSAID use during follow-up	32 (69.57%)	12 (52.17%)	20 (86.96%)

Legend. *There were four patients exposed to a non-TNFi bDMARD; all were also TNFi-exposed. Three of four started the TNFi first and of those, two did not start the non-TNFi until after the second MRI that was used for the change in inflammation assessment

### Interrater reliability

The interrater reliability across the three radiologists was excellent for the SIS status scores (ICC = 0.89) and fair to good for the SSS components’ status scores for erosion (ICC = 0.57), fat metaplasia (ICC = 0.52), and ankylosis (ICC = 0.72). Agreement was poor for the remaining SSS components: sclerosis (ICC = 0.32) and backfill (ICC = 0.33). The relatively low reliability for some of the SSS components was partially due to the low prevalence of reported lesions in this pediatric cohort with only 13 (36.1%) erosion, 6 (23.1%) sclerosis, 2 (5.6%) fat metaplasia, 3 (8.3%) backfill, and 2 (5.6%) ankylosis cases.

### SPARCC inflammation scores

Forty-five subjects (98%) had 2 evaluable SIS studies at least 12 weeks apart. Twenty-three (51%) and 22 (49%) subjects had an SIS of < 2 and ≥ 2 on the baseline scan, respectively. Cases with an SIS ≥2 versus those with SIS < 2 on baseline imaging did not present with a statistically significant difference in clinical disease activity as measured by physician global disease activity assessment (median = 3 [IQR: 2–4] vs 2 [IQR: 2–3], *p* = 0.33). The median SIS at baseline in all patients was 0 (IQR: 0–19) and in those with SIS ≥2 was 19.9 (13.3–28.5), respectively. Nineteen (42.2%), 10 (22.2%), and 16 (35.6%) cases demonstrated improvement, worsening, or no change in the SIS over time, respectively. Subjects whose SIS improved over time had a median decrease in SIS of 20.2 (IQR: 11–27). Subjects whose SIS worsened had a median increase in SIS of 8 (IQR: 4–14.5). Of those with worsening of the SIS from 0 to ≥1 (*N* = 7), one was exposed to a TNFi but was off therapy for nine months preceding the second MRI. Correlation of change in patient-reported pain with change in SIS was weak (r = 0.14).

Unadjusted inflammation change scores are presented in Table 2. The median change in SIS in TNFi exposed and unexposed subjects with a baseline SIS ≥0 was − 20.7 and − 14.3, respectively (*p* = 0.09). Eleven (85%) TNFi-exposed and 8 (89%) TNFi-unexposed subjects with a baseline SIS ≥0 met the SIS MCID of change ≥ − 2.5. Of the patients who met the MCID, 81.8 and 50% of TNFi exposed and unexposed, respectively, had resolution of inflammation. Table 3 shows clinical details of the TNFi unexposed patients who met the SIS MCID. One unexposed subject received TNFi for 67 days but did not meet the protocol-defined definition of TNFi exposure (≥90 days).

**Table 2:** Unadjusted inflammation change scores

	All subjects	TNFi exposed	TNFi unexposed	
	Median SIS Change Score (IQR)			*p*-value
All subjects	0.0 (−14.7, 0.0) [*N* = 45]	−5.2 (−24.7, 0.0) [*N* = 22]	0.0 (− 10.3, 4.0) [*N* = 23]	0.09
Baseline SIS≥2*	−16.8 (−26.0, − 10.3) [*N* = 22]	−20.7 (−26.0, − 11.0) [*N* = 13]	−14.3 (−20.2, −9.3) [*N* = 9]	0.09
Baseline SIS = 0	0.0 (0.0, 4.0) [*N* = 23]	0.0 (0.0, 0.0) [*N* = 9]	0.0 (0.0, 4.0) [*N* = 14]	0.12
	Frequency meeting MCID (%)			p-value
All subjects	19 (42.22%) [*N* = 45]	11 (50.00%) [*N* = 22]	8 (34.78%) [*N* = 23]	0.30
Baseline SIS> 0	19 (86.36%) [*N* = 22]	11 (84.62%) [*N* = 13]	8 (88.89%) [*N* = 9]	0.77

Legend. MRI sequence availability dictated which subjects were evaluated with SIS and SSS detailed scoring. Of the 46 unique subjects, one was missing the necessary sequences to perform SIS detailed scoring. *SPARCC SIS score ≥ 2 is used as a surrogate for MRI-sacroiliitis positive in clinical trials [23]

**Table 3:** Subjects without TNFi exposure who met SIS MCID

Subject	Sex	Time between MRI scans (months)	Pain change score	Baseline SIS	Follow-up SIS	Baseline Erosion	Follow-up Erosion	NSAIDs	cDMARD	TNFi exposure (days)
1	M	8.5	–	34.7	7	11	4.3	None	None	0
2	M	33.8	−4	14.3	0	5.3	5.7	Yes	MTX	0
3	M	22.1	–	3	0	0	0	Yes	SSZ	0
4	M	5.8	–	13.3	4	5	9	Yes	None	0
5	M	36.0	–	30.7	16	0	0	Yes	None	0
6	F	35.7	–	24.7	4.5	0	4.7	Yes	SSZ	0
7	M	7.5	−1	10.3	0	0	0	Yes	None	0
8	F	2.9	−1	28.5	0	10	13	Yes	MTX	67

Legend. M = Male, F=Female, NSAIDs = nonsteroidal anti-inflammatory drugs, csDMARD = conventional disease modifying anti-rheumatic drug; MTX = methotrexate, SSZ = sulfasalazine

Using the ATE model adjusted for baseline SIS, the average effect of TNFi on SIS in all patients was −7.85 (95% CI: − 12.22, −3.49; *p* < 0.01). The ATE for patients with a baseline SIS ≥2 was − 14.50 (95% CI: −21.62, − 7.37; *p* < 0.01) and for those with a baseline SIS < 2 was − 1.27 (95% CI: −4.37, 1.83; *p* = 0.42) (Table 4). Sensitivity analyses with definitions of TNFi exposure as ≥60, ≥120 and ≥ 180 days demonstrated nearly identical results with baseline SIS ≥2 with ATE of − 15.22 (95% CI:-22.10, −8.33; *p* < 0.001), − 15.99 (95% CI: −23.04, − 8.95; *p* < 0.001), and − 14.47 (95% CI: − 18.78, − 10.16; *p* < 0.001), respectively. Sensitivity analysis of patients excluding the 2 patients in the TNFi exposed group who were also exposed to a non-TNFi biologic between MRI studies demonstrated similar results with ATE of − 8.09, − 14.5, and − 1.10 for all patients, patients with baseline SIS ≥2 and baseline SIS < 2, respectively.

**Fig. 1:**
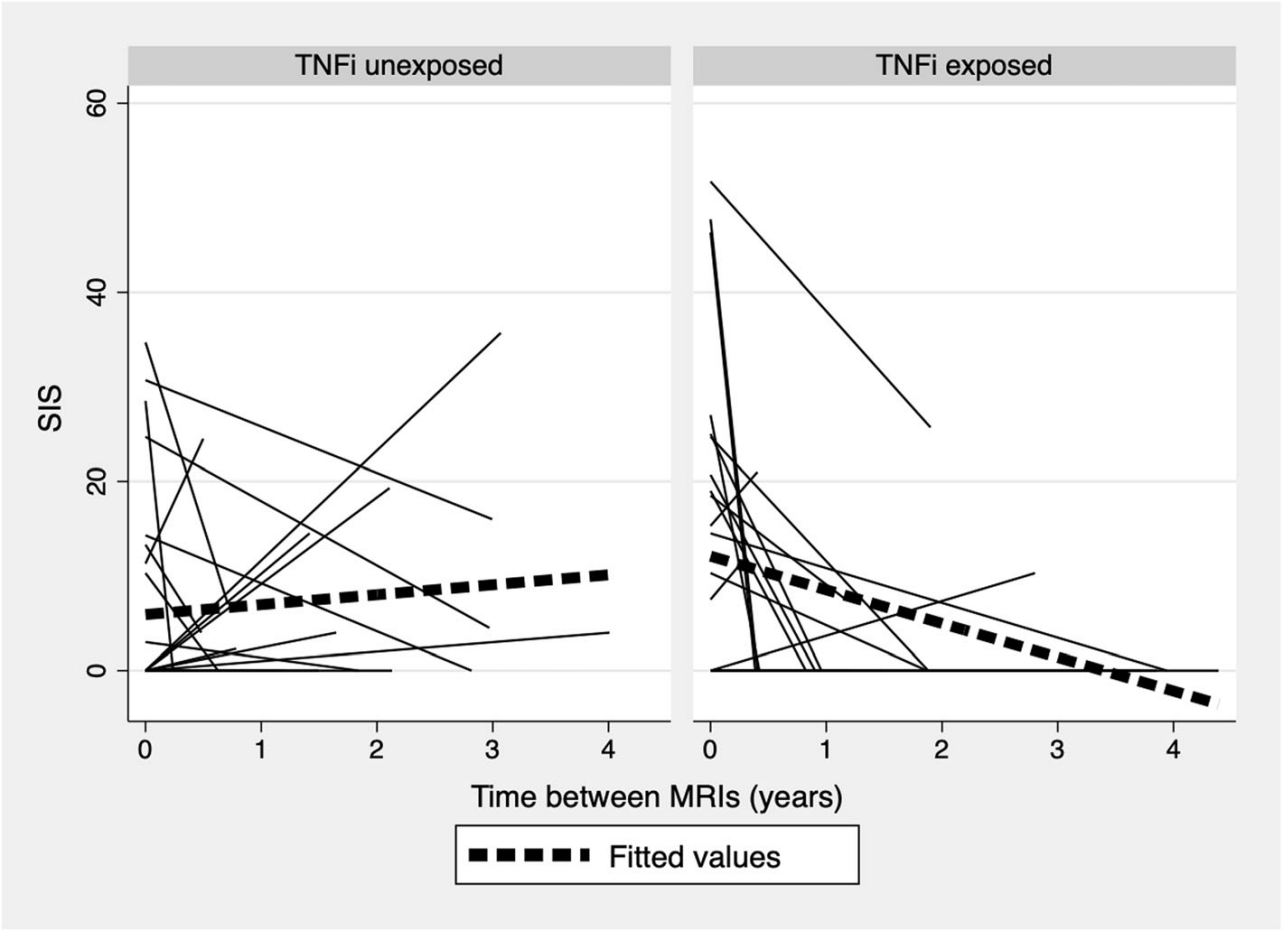
Unadjusted SIS trajectory of all subjects (*N* = 45). Each solid line begins at a subject’s baseline SIS score and ends at their follow-up score. Dotted lines are the overall fitted regression lines. The TNFi exposure was defined as ≥90 days of use between MRIs. SIS = sacroiliac joint inflammation score; TNFi = tumor necrosis factor inhibitor

A spaghetti plot of SIS stratified by TNFi exposure suggests a consistency with these results, demonstrating a negative slope of fitted line in TNFi exposed patients and a positive slope in TNFi unexposed patients (Fig. 1).

**Table 4:** Treatment effects

	N	Average treatment effect (95% CI)	p-value
**SIS**			
All	42	−7.85 (− 12.22, −3.49)	0.00
Baseline SIS≥2*	21	− 14.50 (− 21.62, − 7.37)	0.00
Baseline SIS< 2	21	− 1.27 (− 4.37, 1.83)	0.42
**SSS**			
Erosion	18	0.72 (−1.88, 3.31)	0.588
Sclerosis	18	−0.18 (− 0.93, 0.56)	0.628

Legend. Average treatment effects from TNFi use, adjusting for baseline SIS or SSS lesions scores as appropriate. Exposure defined as ≥90 days of TNFi use. *SPARCC SIS score ≥ 2 is used as a surrogate for MRI-sacroiliitis positive in clinical trials [23]

### SPARCC structural scores

**Fig. 2:**
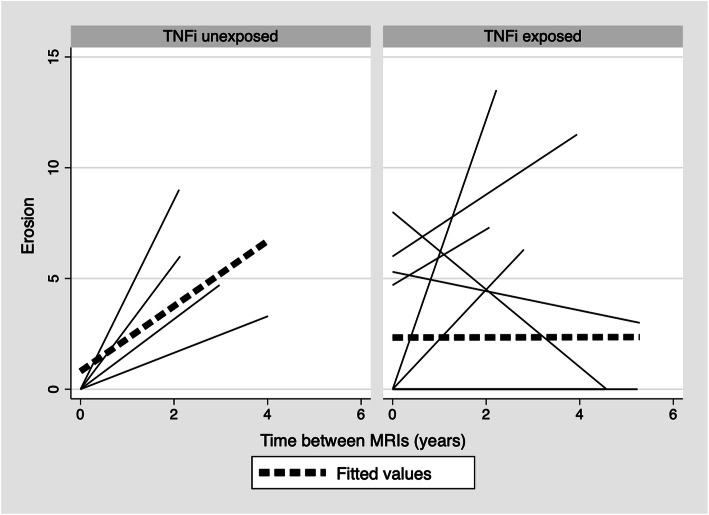
Unadjusted trajectory of SSS erosion in all subjects (*N* = 38). Each solid line begins at a subject’s baseline SSS erosion score and ends at their follow-up score. Dotted lines are the overall fitted regression lines. The TNFi exposure was defined as ≥90 days of use between MRIs. SSS = sacroiliac joint inflammation score; TNFi = tumor necrosis factor inhibitor

Eighteen subjects, 14 TNFi exposed and 4 TNFi unexposed, with two MRI studies at least 2 years apart were included in the analysis of SSS lesions. Four (28.6%) TNFi exposed and 0 (0.0%) TNFi unexposed patients had erosion scores > 0 at baseline. Of the four patients with a baseline erosion score > 0, 2 (50.0%) demonstrated improvement and 2 (50.0%) demonstrated worsening in the erosion score over time. In cases where the baseline erosion score was zero, all 4 (100.0%) of the unexposed patients and 2 (20.0%) of the TNFi exposed patients had an erosion score > 0 on follow-up imaging. Unadjusted median SSS erosion change scores in TNFi unexposed subjects signaled a statistically significant (*p* = 0.03) overall worsening in erosion score (5.3 IQR: 4.0, 7.5) whereas the TNFi exposed subjects experienced very little change in erosion score (0.0 IQR, 0.0, 2.6). As mentioned previously, all four (100.0%) of the TNFi unexposed patients worsened. In the TNFi exposed group, 4 (28.6%) worsened, 8 (57.1%) stayed the same, and 2 (14.3%) improved. Spaghetti plots of SSS erosion trajectory stratified by TNFi exposure suggest a consistency with these results, demonstrating a positive slope (worsening) in TNFi-unexposed subjects and a relatively flat slope (no change) in TNFi exposed subjects for the respective fitted lines (Fig. 2). Using the ATE model adjusted for baseline erosion score, the average effect of TNFi on SSS erosion was not significant and was 0.72 (95% CI, − 1.88, 3.31; *p* = 0.59; Table 4). Sensitivity analysis of patients excluding the 3 patients in the TNFi exposed group who were also exposed to a non-TNFi biologic between MRI studies demonstrated similar results with ATE of 1.20 (95% CI, − 1.60, 4.00; *p* = 0.40).

Three (21.4%) TNFi exposed patients and 0 (0.0%) TNFi unexposed patients had sclerosis scores > 0 at baseline. Of these three cases where the baseline sclerosis score was > 0, 2 (66.7%) demonstrated improvement and 1 (33.3%) demonstrated worsening in the sclerosis score over time. In cases where the baseline sclerosis score was zero, none (0.0%) of the TNFi unexposed patients and one of the TNFi exposed patients had a sclerosis score > 0 at the time of follow-up imaging. The median unadjusted SSS sclerosis change scores in TNFi exposed and unexposed subjects were both 0.0 (95% CI: 0.0, 0.0). All four (100.0%) of the TNFi unexposed patients had unchanged zero sclerosis scores during follow-up. In the TNFi exposed group, 2 (14.3%) worsened, 10 (71.4%) stayed the same, and 2 (14.3%) improved. Using the ATE model adjusted for baseline sclerosis score, the average effect of TNFi on SSS sclerosis was not significant and was − 0.18 (95% CI:-0.93, 0.56; *p* = 0.63; Table 4). Sensitivity analysis of patients excluding the 3 patients in the TNFi exposed group who were also exposed to a non-TNFi biologic between MRI studies demonstrated similar results with ATE of − 0.09 (95% CI: − 0.92, 0.74; *p* = 0.82).

Models were not run on ankylosis, backfill, or fat metaplasia due to the small number of lesions in the cohort.

## Discussion

Our objective was to describe MRI changes over time in inflammatory and structural lesions at the sacroiliac joints in children with SpA exposed and unexposed to TNFi. Data on longitudinal changes at the pediatric SIJ are sparse and greatly needed. In our small, uncontrolled cohort, we observed that differences exist in SIJ lesions between TNFi-exposed and TNFi-unexposed patients. Raw, median inflammation change scores showed more improvement in TNFi-exposed patients that approached statistical significance and TNFi use was also associated with a significant average treatment effect in inflammation on MRI at the sacroiliac joints in juvenile SpA. Unadjusted median erosion change scores were significantly higher in TNFi unexposed versus exposed subjects but in the ATE model adjusted for baseline erosion score the effect of TNFi was not significant. Although ATE model is most appropriate for randomized trials, here we applied this model only for purpose of exploration rather than making formal hypothesis testing. Stabilization of the erosion score without progression is also a positive clinical outcome. These results support the use of change in SIS and SSS as objective tools to assess response to biologics in effectiveness and efficacy studies.

Lesion detection reliability across raters was in line with expected thresholds for lesions that were not rarely reported in this cohort. Sclerosis and backfill had the lowest levels of agreement which can most likely be attributed to the low prevalence reported by any rater. The remaining SPARCC SIS and SSS components met the literature reported agreement of fair to excellent [20–22].

Surprisingly, the majority of TNFi unexposed subjects with a baseline SIS ≥0 met the SIS minimal clinically important difference (MCID ≥2.5), and this will need to be explored in future studies. Half (4 of 8) of unexposed patients achieving the MCID demonstrated total resolution of inflammation on the follow-up study. One of these subjects had mild inflammation at baseline (SIS = 3) and one was actually TNFi exposed but did not meet the study’s definition of exposure (≥90 days). The results in the unexposed group underscore the importance of ascertaining the natural history of inflammatory change in the pediatric SIJ as this is currently unknown. Based on clinical experience and studies in adults, TNFis provide symptomatic relief to patients with sacroiliitis, but perhaps not all patients require biologic therapy for resolution to occur. Patients in this study were often treated with an NSAID if they weren’t also on a biologic. There is limited evidence from studies in adults with ankylosing spondylitis that NSAIDs, particularly celecoxib, may play a role in slowing progression of structural damage [25, 26] though data from a more recent study did not confirm these findings [27]. The role of monotherapy with NSAIDs has not been evaluated in children with sacroiliitis and is unlikely to be studied since the most recent ACR guidelines strongly recommend initiation of a biologic over NSAID monotherapy [28]. Future well-designed studies need to ascertain which patients are most likely to have persistent inflammation and would benefit from biologic initiation.

Validated measures to assess progression of sacroiliitis include the New York (NY) criteria [19], the SPARCC sacroiliac joint inflammation score [19], and the SPARCC sacroiliac joint structural score. The NY criteria are used on radiographs, only assess structural damage, and have moderate interrater reliability [29]. Despite its limitations, the NY criteria remain the gold standard for classification of ankylosing spondylitis and assessment of radiographic progression in clinical trials [30]. There are preliminary data signaling that use of a biologic compared to non-biologic agent may slow radiographic progression, but the effect is only seen when the gap between radiographs is quite long (approximately 4 years) and serial radiographs cannot inform about change in active inflammation [31]. The SPARCC SIS is being increasingly used to assess response at the sacroiliac joints in clinical trials and offers an objective measure of change that can be assessed over a much shorter time period than radiographs. Several studies have demonstrated the ability to detect significant changes in the SPARCC SIS in as little as 12 and 16 weeks after initiation of a biologic in adults with non-radiographic SpA [12, 32–34]. Our results support the use of the SIS in the pediatric population with axial arthritis as well. To date, there have been no pediatric randomized clinical trials of biologic agents for axial arthritis that include evaluation of serial imaging. Objective imaging measures of response to these novel agents are critical to assess since complaints of inflammatory back pain by history and pain to palpation have relatively low positive predictive value in the pediatric population [10]. Use of these subjective measures instead of objective measures like the SPARCC SIS may increase the likelihood of missing significant findings.

One limitation of this study was the rarity of structural lesions other than erosion. As such, we were unable to run our models to assess for the association of cumulative TNFi exposure with change over time in most of these lesions. Our inability to show significant association of TNFi with change in erosions and sclerosis scores using the adjusted ATE model may be because we were underpowered to detect the association or perhaps, as suggested by prior work [15], TNFis do not halt structural progression. However, it is notable that in Fig. 2, the positive slope of the fitted line indicates worsening for erosion in TNFi unexposed cases, while the flat slope of the fitted line in the TNFi exposed patients indicates that the progression, on average, is at least slowed. A larger sample size and a cohort with a greater prevalence of structural damage and longer disease duration and/or follow-up is needed to further evaluate the associations of cumulative TNFi exposure and change over time in these lesions.

The retrospective observational study design raises a few additional limitations that should be considered. First, there was variability in the imaging protocols performed at each of the three institutions and over time within institutions. The ability to score the SPARCC SIS and SSS is primarily dependent on the presence of coronal oblique T1W and fluid-sensitive sequences of the sacroiliac joints; since these sequences were present in all studies per our inclusion criteria, the validity of our results are not likely to be impacted by minor imaging protocol differences. All studies were performed on high field-strength magnets (1.5 T or 3.0 T). Second, there is the potential for confounding by indication whereby patients in the TNFi-exposed group had more severe disease. However, there were no statistically significant differences in clinical features between groups and, if residual unmeasured confounders were present, this would bias the study toward the null hypothesis of no difference in change in SIS or SSS. Third, the time between imaging studies and duration and magnitude of TNFi exposure varied as the studies were ordered as per clinical care. There is likely also selection bias introduced into the cohort based on which patients are more likely to have a repeat MRI and receive more aggressive treatment. In the collective authors’ experience, insurance will often not pay for an MRI to confirm resolution of inflammation or in the absence of symptoms, so most repeat MRIs are performed in patients who are having symptoms. That practice, however, would bias the results towards the null or finding no difference in those exposed and unexposed to TNFi therapy. Another limitation that could result in a diminished observed effect in our analysis is that it was difficult to account for treatment regimen non-compliance. Some physician notes detailed incidents of missed doses for a variety of reasons and other patients may have intentionally not been taking their medication as prescribed. The effect on the sacroiliac joints of these gaps in therapy or non-adherence to treatment plan is unknown. Lastly, several subjects in the TNFi exposed group received more than one TNFi sequentially between eligible MRI studies, most likely indicating poor response to therapy again biasing our results towards the null and making the ability to detect a difference between the two groups even more impressive.

## Conclusion

In summary, we describe how MRI lesions in the SIJ change under different treatment conditions. TNFi use was associated with a significant average treatment effect in inflammation at the sacroiliac joints in juvenile SpA. We also observed a trend, albeit insignificant, in TNFi exposure and a halted progression in the SSS erosion score for studies done at least two years apart. Incorporation of the SIS and SSS into not only clinical trials but also effectiveness studies should be strongly considered as they provide feasible and responsive means of objectively assessing response over a short period of time. An enhanced understanding of the magnitude and rate of response to TNFi in children will help to inform the design of clinical trials.

## Data Availability

The data that support the findings of this study are available from the corresponding author but restrictions apply to the availability of these data, which were used under data use agreements for the current study, and so are not publicly available. Data are however available from the authors upon reasonable request and with permission of all sites contributing data.

## Abbreviations

SpA: spondyloarthritis
TNFi: tumor necrosis factor inhibitor
MRI: magnetic resonance imaging
ATE: average treatment effects
SPARCC: Spondyloarthritis Research Consortium of Canada
SSS: sacroiliac joint inflammation score SIS and structural score
DICOM: Digital Imaging and Communication in Medicine
STIR: Short-tau inversion recovery
BME: bone marrow edema
ICCs: Intraclass correlation coefficients
IQR: Interquartile range
CI: Confidence interval
